# Effect of docosahexaenoic acid on in vitro growth of bovine oocytes

**DOI:** 10.1002/rmb2.12403

**Published:** 2021-07-12

**Authors:** Shuta Nagata, Kaoru Tatematsu, Hitoki Yamaguchi, Yuki Inoue, Keisuke Tanaka, Hidetaka Tasaki, Koumei Shirasuna, Hisataka Iwata

**Affiliations:** ^1^ Department of Animal Science Tokyo University of Agriculture Atsugi Japan; ^2^ NODAI Genome Research Center Tokyo University of Agriculture Tokyo Japan; ^3^ Assisted Reproductive Technology Center Okayama University Okayama Japan; ^4^ Graduate School of Environmental and Life Science Okayama University Okayama Japan

**Keywords:** ATP, DHA, lipid, oocyte growth

## Abstract

**Purpose:**

The present study investigated the effects of docosahexaenoic acid (DHA) on the growth of bovine oocytes.

**Methods:**

Oocytes and granulosa cell complexes (OGCs) were collected from early antral follicles (0.4‐0.7 mm) on the surface of ovaries harvested from a slaughterhouse. The OGCs were cultured with 0, 1, and 10 μmol/L docosahexanoic acid (DHA) for 16 days.

**Results:**

Antrum formation of the OGCs and the number of granulosa cells (GCs) surrounding the oocytes were comparable among groups, whereas supplementation of 0.1 μmol/L of DHA significantly improved oocyte growth. Oocytes grown with DHA had a higher fertilization rate, acetylation levels of H4K12, and ATP contents, as well as a lower lipid content compared with those grown without DHA. In addition, GCs surrounding OGCs grown with DHA had low lipid content compared with vehicle counterparts. Furthermore, when GCs were cultured in vitro, DHA increased ATP production, mitochondrial membrane potential, and reduced lipid content and levels of reactive oxygen species. RNA‐seq of GCs revealed that DHA increased *CPT1A* expression levels and affect genes associated with focal adhesion, oxidative phosphorylation, and PI3K‐AKT etc

**Conclusion:**

The results suggest that DHA supplementation affects granulosa cell characteristics and supports oocyte growth in vitro.

## Abstract 2

Docosahexaenoic acid improves growth of oocytes derived from early antral follicles.

## 1 INTRODUCTION

Oocyte growth occurs in follicles, and follicular fluid (FF) is the sole environment for developing oocytes and granulosa cells (GCs). FF contains a myriad molecules that support oocyte growth, and fatty acids are a major component. Polyunsaturated fatty acids (PUFAs) consist of approximately half of the fatty acids in human FF,^1^ and n‐3 PUFA, α‐linolenic acid (ALA, C18:3 n‐3), eicosapentaenoic acid (EPA, C20:5 n‐3), and docosahexaenoic acid (DHA, C22:6 n‐3) are crucial, because mammals cannot produce ALA, a precursor of the other PUFA, and the only source of PUFA is food. Six‐week omega 3 dietary intake improved embryo development markers in humans; shorter time to complete four cell cycle and synchrony for the third cell cycle, and high concentration of serum ω3PUFA were associated with the high clinical outcomes after assisted reproductive technology.^2^, ^3^ In this context, the beneficial effects of DHA on cow reproduction have been reported. For example, DHA concentration is higher for follicular fluid (FF) of dominant follicles compared with those of subordinate follicles in both cows and heifers.^4^ In addition, supplementation of cattle with fish oil or algae containing DHA increased DHA concentration in plasma and improved the number and size of follicles as well as reproduction performance.^5^, ^6^, ^7^ Although the causal relationship between good reproductive performance and DHA is unclear, some pioneering studies have reported that supplementation of the in vitro maturation medium of oocytes with DHA improves the developmental competence of oocytes, cleavage of embryos, and the quality of blastocysts in cows and pigs.^8^, ^9^ In addition, Elis et al^10^ reported that supplementation of the maturation medium of oocytes with DHA decreased lipid content in both oocytes and cumulus cells, and supplementation with the fatty acid receptor FFAR4 agonist TUG‐891 also improved oocyte developmental competence. However, in vitro maturation of bovine and porcine oocytes takes only one and two days, respectively. In large mammals such as pigs and cows, oocyte growth takes longer periods, but no study has addressed the effect of DHA on oocyte growth. In the present study, oocytes derived from early antral follicles were cultured for 16 days, and the effect of DHA on in vitro oocyte growth was examined.

## 2 MATERIALS AND METHODS

### 2.1 Ethical approval

Collection of ovaries from a slaughterhouse for experimental use was approved by the Committee for the Care and Welfare of Experimental Animals at Tokyo University of Agriculture.

### 2.2 Medium and chemicals

All chemicals were purchased from Nacalai, unless otherwise stated. Docosahexaenoic acid ≥98% (DHA) (D2534, Sigma‐Aldrich) was diluted in ethanol (1000 times the final concentration), and the control group was added with the same volume of vehicle. The concentration of DHA is determined basis on the concentration of DHA in bovine FFs (0.43‐1.0 μg/mL:1.3‐3 μmol/L)^4^ and a report studying the effect of DHA on bovine oocyte maturation (1‐100 μmol/L).^5^

The medium used for oocyte GCs complexes (OGCs) (IVG medium) was α‐minimum essential medium (αMEM, Sigma‐Aldrich) supplemented with 1 μg/mL 17b‐estradiol, 0.02 mAU/mL follicle‐stimulating hormone (Kawasaki Mitaka), 11 mmol/L glucose, 0.9 mmol/L pyruvic acid, 0.05 μmol/L dexamethasone, 50 µg/mL ascorbic acid, 55 µg/µL l‐cysteine, 2% polyvinylpyrrolidone‐90K (Sigma‐Aldrich), 4 mmol/L hypoxanthine (Sigma‐Aldrich), 5% fetal calf serum (FCS), and antibiotics (100 IU/mL penicillin, 0.1 μg/mL streptomycin, and 50 μg/mL gentamicin). TCM‐199 medium (Gibco BRL) supplemented with 10% FCS, 0.06 mmol/L pyruvic acid, 10 mmol/L taurine, and antibiotics was used for in vitro maturation (IVM) of oocytes. The medium used for in vitro fertilization (IVF) and culture (IVC) was synthetic oviductal fluid (SOF),^11^ with minor modifications.^12^ IVF‐SOF contained 5 mg/mL of fatty acid‐free bovine serum albumin (BSA) and 10 IU/mL heparin (Sigma‐Aldrich). IVC‐SOF contained essential and nonessential amino acids (Sigma‐Aldrich), 1.5 mmol/L glucose, and 1% FCS. The medium used for in vitro culture for GCs was TCM‐199 (Gibco) supplemented with 5% FCS. Incubation was performed at 38.5℃ in an atmosphere of 5% CO_2_ and saturated humidity.

### 2.3 OGCs collection and in vitro culture of the OGCs

Ovaries were collected at a local slaughterhouse and transported to the laboratory (at approximately 25℃ in PBS containing antibiotics) within 4 hours. The ovarian cortical tissues were excised from the ovarian surface under a stereomicroscope, and OGCs were collected from early antral follicles (EAFs, 0.4‐0.7 mm in diameter). OGCs were observed under a digital microscope (Keyence) to measure their diameter, and OGCs surrounded by thick compact GCs and oocytes (90‐100 μm in diameter) were selected for experiments. As described in a previous report,^13^ OGCs were individually cultured for 16 days in 200 μL of IVG medium in 96‐well plates with polyacrylamide gels at the bottom. The number of OGCs forming an antrum was counted every 2 days. Half of the medium was replaced with fresh medium on days 4, 8, and 12. At the end of the culture period (16 days), OGCs with an antrum cavity were further analyzed. Almost all OGCs without antrum contained degenerated oocytes.

### 2.4 Measurement of diameter of oocytes grown in vitro

Oocytes were denuded from surrounding cells. The diameter of the ooplasm (horizontal and vertical diameters) was measured using a digital microscope (Keyence). The average of the two values was calculated and reported as the diameter.

### 2.5 Measurement of the number of GCs consisting a OGCs grown in vitro

GCs were detached from oocytes and dispersed by vigorous pipetting in a cell‐dispersion cocktail (Accumax; Innovative Cell Technologies, Inc). The total cell number was calculated using a hemocytometer to obtain the average GC number per OGC.

### 2.6 Measurement of lipid content in oocytes and surrounding GCs

Granulosa cells and oocytes were separated from each OGC. Lipid content was determined by Nile red staining (Wako), as described previously.^14^ Briefly, oocytes were incubated for 10 minutes in PBS containing 10 μg/mL Nile Red. Fluorescence images of the oocytes were captured using a fluorescence microscope (Keyence), and the fluorescence intensity of whole oocytes was measured using ImageJ software (National Institutes of Health, Bethesda, MD, USA). In addition, GCs were enzymatically dispersed as described above and stained with Nile red and Hoechst 33342. GCs were observed under a fluorescence microscope (Leica), and the images were captured to obtain the ratio of the fluorescence intensity of Nile red to that of Hoechst. In addition, the GCs were subjected to flow cytometry using a NovoCyte Flow Cytometer (2000R, ACEA Biosciences, Inc) in FL2 channel (excitation laser 488 nm, emission filter 572/28 nm), followed with data analysis with NovoExpress Software.

### 2.7 Detection of acetylated H4K12 by fluorescence immunostaining

Oocytes were fixed in 4% paraformaldehyde for 1 day and subjected to immunostaining. Immunostaining was performed as previously reported.^15^ The primary and secondary antibodies used for this procedure were rabbit polyclonal anti‐H4K12 (1:200; Novus International Saint Charles) and fluorescein‐conjugated goat anti‐rabbit IgG (1:500; Cell Signaling Technology Inc), respectively. Oocytes were mounted onto glass slides with an antifade reagent containing DAPI (ProLong™Gold antifade reagent with DAPI; Invitrogen). Oocytes were observed under a Leica DMI 6000 B microscope using LAS AF software (Leica), and fluorescent intensities of oocytes were quantified using ImageJ software (NIH).

### 2.8 Measurement of ATP in oocytes

At the end of IVG, oocytes were denuded from GCs, and ATP content was determined by measuring the luminescence generated in an ATP‐dependent luciferin‐luciferase reaction (ATP assay kit; Toyo‐Inc), as described previously.^16^ Each sample was prepared by adding individual oocytes to 50 μL of distilled water.

### 2.9 In vitro maturation and fertilization

All OGCs with an antrum cavity were selected, and oocytes with 2‐3 GC layers were removed. The oocytes were cultured in IVM for 24 hours, and the frequency of oocytes at metaphase 2 stage, or fertilization rate, followed by IVF, was determined. For IVF, the oocytes were co‐incubated with thawed semen from a Japanese black bull for 5 hours, and then the oocytes were subsequently cultured for 13 hours to examine the fertilization rate. The semen was washed with a 45%‐60% Percoll solution (Amersham Biosciences) to create a discontinuous gradient for centrifugation (800 *g* for 10 minutes). The final sperm concentration in IVF medium was 1 × 10^6^ cells/mL. To evaluate the fertilization rate, oocytes were denuded from the surrounding GCs, and the oocytes were transferred into aceto‐alcohol (ethanol:acetic acid = 3:1) for 3 minutes, and the number of pronuclei was examined under a stereomicroscope (Olympus). Oocytes with two clear pronuclei were determined to be normally fertilized oocytes. Oocytes with over three pronuclei or one pronucleus were determined to be abnormal fertilized oocytes. Maturation and fertilization rates were examined four and five times, respectively.

### 2.10 GC collection and in vitro culture of the GCs

Granulosa cells were aspirated from antral follicles (3‐5 mm in diameter) on at least 20 cows using a syringe connected to an 18‐gauge needle and washed in culture medium. Cellular debris was removed from the cellular pellets using a filter (pore size 60 μm) and cultured on plastic dishes (60 mm NUNC) for 24 hours. As the GCs contained dead cells, the surviving GCs attached to the plastic plate were collected by Accumax (Innovative Cell Technologies) treatment. GCs were seeded in 96‐well plates (Cat. No 353072; BD Biosciences) at a final concentration of 200 000 cells/mL (100 μL/well). The next day (2 days after collection), the morphology and density (70%–80% confluent) of the GCs were examined under a microscope (Olympus), and the culture medium was changed with the medium containing DHA or vehicle.

### 2.11 Lipid content and ATP content in GCs cultured in vitro

Granulosa cells cultured on glass bottom plates (Thermo Fisher) at a final concentration of 200 000 cells/mL (100 μL/well) for 48 hours were stained with Nile Red as described above and their nuclei were counterstained with Hoechst 33342. Fluorescent images of GCs were captured using a fluorescence microplate reader (Spark 10 M, Tecan), and the ratio of Nile Red/Hoechst 33342 was determined. To determine the ATP content, GCs in each well were frozen and thawed three times with 100 µL water, and water was collected from each well following vigorous pipetting. This water (50 μL) was used to determine the ATP content by measuring the luminescence generated during an ATP‐dependent luciferin‐luciferase reaction using an ATP assay kit (Toyo‐Inc). In addition, the remaining half of the water was used for DNA extraction, and the copy number of nucleic DNA was determined by real‐time PCR as described below. Thereafter, the ATP content of 10 000 GCs was calculated.

### 2.12 Measurement of mitochondrial and nucleic DNA copy number in GCs

Granulosa cells cultured on 96‐well plates were lysed in 50 μL of lysis buffer (20 mmol/L Tris, 0.4% proteinase K, 0.9% Nonidet‐P40, and 0.9% Tween 20) followed by incubation at 55℃ for 30 minutes and then at 95℃ for 5 minutes. The nuclear DNA (nDNA) and mitochondrial (mtDNA) copy number in GCs were determined using real‐time PCR targeting the bovine mitochondrial genome and a single‐copy nuclear gene. PCR was performed using a CFX Connect™ real‐time PCR detection system (Bio‐Rad). The PCR primer set was designed using Primer3Plus (https://www.bioinformatics.nl/cgi‐bin/primer3plus/primer3plus.cgi). Primers for nDNA were 5′‐ttccactctgcacagtagcg‐3′ and 5′‐cccttactggttgtggcact‐3′, targeting a one‐copy sequence of 83 bp (NC_037334.1). The primers for Mt‐DNA were 5′‐acccttgtacctttgcat‐3′ and 5′‐tctggtttcgggctcgttag‐3′ targeting a mitochondrial genome sequence of 81 bp (NC_006853.1). The PCR conditions were as follows: initial denaturation at 95℃ for 1 minute, followed by 40 cycles at 98℃ for 5 seconds and 60℃ for 10 seconds. A standard curve was generated for each run using 10‐fold serial dilutions representing the copy number of the external standard. The external standard was the PCR product of the corresponding gene cloned into a vector using the Zero Blunt TOPO PCR cloning kit (Invitrogen), which was sequenced before use. DNA copy number in the standard was calculated using the concentration of DNA, molecular weight of the vector, and Avogadro's number. The amplification efficiency in all trials was >1.98. Thereafter, mtDNA per nDNA was calculated to obtain the mitochondrial copy number per granulosa cell.

### 2.13 Measurement of mitochondrial membrane potential (MMP) and ROS content in GCs

Granulosa cells cultured in a glass bottom plate (Thermo Fisher) were stained with a combination of MitoTracker Orange and Green or a combination of Cell ROX and Hoechst 33342. Fluorescence intensity was measured using a fluorescence microplate reader (Tecan), and the fluorescence intensity of MitoTracker Red and Cell ROX was divided by that of Mito‐tracker Green and Hoechst 33342, respectively.

### 2.14 RNA‐seq of GCs cultured with or without DHA

Granulosa cells were collected and cultured with or without DHA for 2 days, as described in Section 2.9 and GCs’ RNA was extracted. RNA extraction was conducted using the RNAqueous^®^ Kit (Life Technologies), and three batches of RNA were produced using differential ovary series. RNA quality was confirmed using an Agilent 2100 Bioanalyzer (Agilent Technologies), and cDNA libraries were prepared using the NEBNext Ultra II RNA Library Prep Kit (New England BioLabs). Library quality and quantity were determined using the Agilent 2100 Bioanalyzer and KAPA Library Quantification Kit (KAPA Biosystems), respectively. A multiplexed library was sequenced as 75 bp fragments (single‐end reads) on a NextSeq 500 platform (Illumina). Sequence data were submitted to the DDBJ Sequence Read Archive under accession number DRA011619 (https://www.ddbj.nig.ac.jp/index‐e.html).

Quality control, data cleaning, gene expression analysis, and identification of differentially expressed genes (DEGs) were performed using the CLC Genomics Workbench 20.0.4 (Qiagen). Raw read data were trimmed for adapter sequences and then filtered under default settings: (a) the quality fraction was limited to 0.05, (b) reads with more than 2 ambiguous bases were discarded, (c) 3 bases of 5′ terminal nucleotides were removed, and (d) adapters were removed automatically by CLC. The clean read data were aligned randomly to a reference Bos taurus genome sequence (ARS‐UCD1.2/GCA_002263795.2, https://asia.ensembl.org/Bos_taurus/Info/Index) to calculate gene expression levels with the following parameters: (a) maximum number of allowed mismatches was 2, (b) maximum number of allowed insertions and deletions was 3, (c) minimum length and similarity fraction was 0.8, and (d) the maximum number of hits for reads was 10 (default). The expression value for each gene was calculated as reads per kilobase of exon model per million mapped reads (RPKM).

Differentially expressed genes were determined using the following criteria: *P* < .05. These processes were performed according to the manual of the CLC Genomics Workbench. Significant Kyoto encyclopedia of genes and genomes (KEGG) pathways enriched by DEGs were determined using DAVID v.6.8 (https://david.ncifcrf.gov/summary.jsp).

### 2.15 Experimental design

First, the effects of various concentrations of DHA on oocyte growth were examined. Ten OGCs were incubated with 0, 1, or 10 μmol/L DHA for 16 days, at the end of culture period oocytes, and GCs were obtained from OGCs forming antrum. The diameter of the oocytes, the number of GCs surrounding the oocytes, and the maturation rate of the oocytes following IVM were examined. The experiment was repeated 9 times. Next, 15 OGCs were cultured with 0 and 1 μmol/L DHA for 16 days and the experiments were repeated 5 times. And the fertilization ability of all oocytes grown in vitro was examined. In the third experiment, 10 OGCs were cultured with 0 and 1 μmol/L DHA for 16 days and randomly selected oocytes were examined for their lipid and ATP content as well as the acetylation levels of H4K12. Furthermore, lipid levels in the GCs were examined. The experiment repeated 7 times. In the fourth experiment, GCs collected from antral follicles were cultured in vitro and the effect of DHA (1 μmol/L) on ATP, MMP lipid, reactive oxygen species (ROS), and mitochondrial copy number were examined. Finally, the effect of DHA on the gene expression of GCs was examined using RNA‐seq.

### 2.16 Statistical analysis

All measurement data are presented as the mean ± standard error of the mean (SEM), and all data were analyzed using the Kolmogorov‐Smirnov test followed by Student's *t* test, and nonparametric data were analyzed using the Mann‐Whitney *U* test. The data among the three groups (DHA, 0, 1, and 10 μmol/L) were analyzed using analysis of variance (ANOVA), followed by Tukey's post hoc test. Maturation and fertilization rate of oocytes were analyzed using Chi square test. Statistical significance was set at *P* < .05. Statistical analysis and calculation of correlation coefficients were conducted using Bellcurve for Excel.

## 3 RESULTS

**FIGURE 1:**
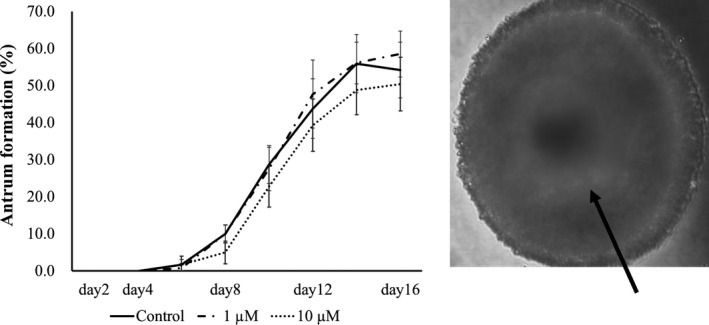
Antrum formation of OGCs cultured with or without DHA. OGCs were cultured for 16 d, and the ratio of OGCs forming antrum was examined. A, Rate (%) of antrum formation during the culture period. B, A representative picture of OGCs forming antrum. Arrow indicates antrum cavity

**FIGURE 2:**
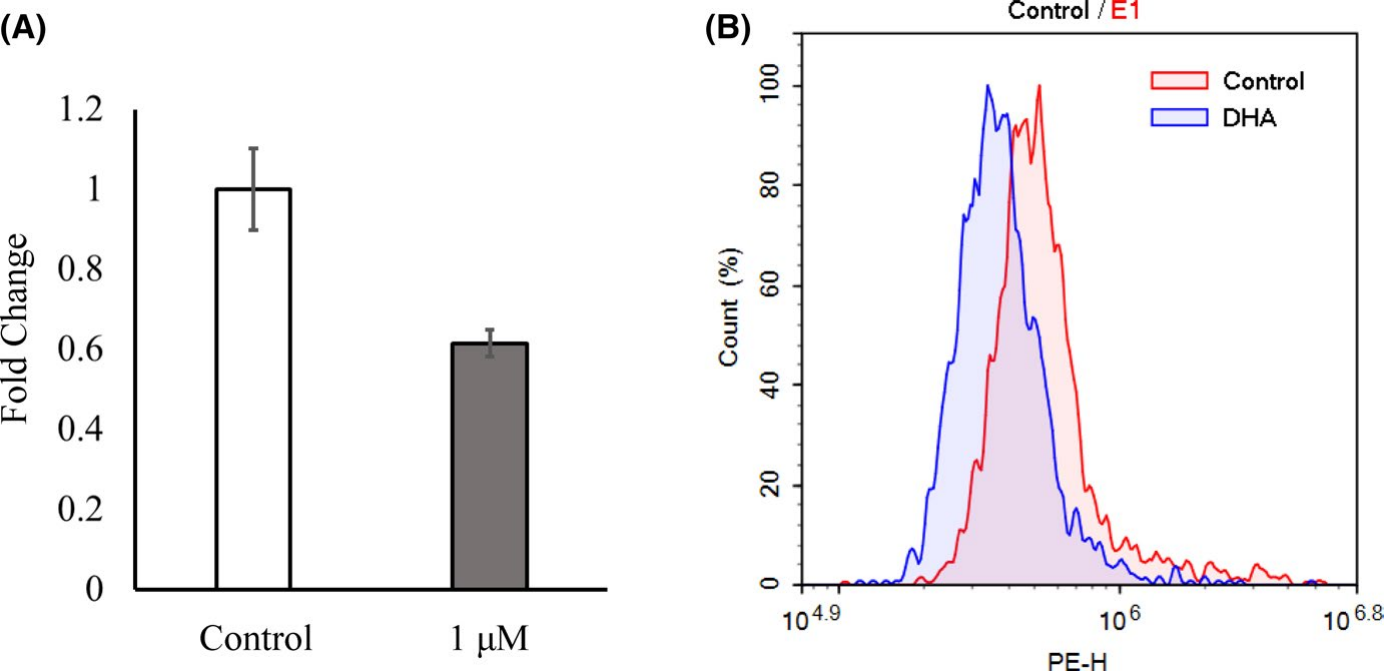
A, Lipid content in granulosa cells (GCs) derived from OGCs treated with or without DHA. Fluorescent intensity of Nile red per Hoechst staining of GCs. Average of control (vehicle, trial number 7) was defined as 1.0. B, GCs stained with Nile red were examined using a flow cytometer [Colour figure can be viewed at wileyonlinelibrary.com]

When OGCs were cultured for 16 days, they formed an antrum‐like cavity surrounding the oocytes, and the rate of antrum formation did not differ among groups (vehicle, 1, and 10 μmol/L DHA) (Figure 1). The number of GCs consisting of OGCs did not differ among the groups, whereas 1 μmol/L DHA significantly increased the diameter of oocytes compared with the vehicle control (Table 1). Half of the oocytes grown with 1 μmol/L DHA reached metaphase 2 stage, but the value did not significantly differ between groups. Oocytes grown with 1 μmol/L DHA had higher fertilization ability with significantly higher total fertilization rate (73.6%) compared with those cultured with vehicle (Table 2). Oocytes grown with 1 μmol/L DHA had higher acetylation levels of histone H4K12 and tended to have higher ATP content (*P* = .08) (Table 3), whereas lipid content in the oocytes was significantly lower than that in oocytes grown with the vehicle. Supplementation of medium with 1 μmol/L DHA decreased the lipid content in the GCs surrounding oocytes (Figure 2A), and the reduction in lipid content was confirmed by FACS (Figure 2B).

**TABLE 1:** Effect of DHA on growth and nuclear maturation of oocytes and number of granulosa cells

DHA (µmol/L)	Diameter of oocytes and number of GCs					Oocytes at M Ⅱ stage			
	No. OGCs	Trial no.	OGCs with AF	Diameter of oocytes	Number of GCs	Trials no.	No. of OGCs	No. of oocytes	M Ⅱ (%)
0	50	5	20	118.8 ± 0.9a	52 435 ± 6918	4	39	26	12 (46.2)
1	50	5	27	122.0 ± 1.0b	66 323 ± 5923	4	40	28	14 (50.0)
10	50	5	17	120.0 ± 1.2ab	63 714 ± 7192	4	40	26	9 (34.6)

Note

Ten oocyte granulosa cells complexes (OGCs) were cultured in medium containing vehicle (ethanol), 1 and 10 μmol/L DHA for 16 d. In the first 5 trials, OGCs forming antrum were selected, and diameter (μm) of in vitro grown oocytes and number of GCs surrounding the oocyte was examined. In the next 4 trials, maturation rate of the oocytes following in vitro maturation was examined. a,b: *P* < .05.

**TABLE 2:** Effect of DHA treatment on fertilization ability of oocytes grown in vitro

DHA (µmol/L)	No. of OGCs	Trials no.	No of oocytes	Number of fertilized oocytes (%)		
				Total	Normal	Abnormal
0	75	5	40	19 (47.5)^a^	16 (40.0)	3 (7.5)
1	75	5	44	33 (75.0)^b^	25 (56.8)	8 (18.2)

Note

Fifteen oocyte granulosa cell complexes (OGCs) were cultured in medium containing vehicle (ethanol) and 1 μmol/L DHA for 16 d, and the rate of fertilization (total rate of oocytes fertilized, normally fertilized, and abnormal fertilized) was examined. a,b: *P* < .05.

**TABLE 3:** Effect of DHA in culture medium on acetylation levels of H4K12, amount of lipid, and ATP content in oocytes grown in vitro

DHA con. (µmol/L)	No. of OGCs	No. of oocytes	Levels of H4K12 acetylation	No. of oocytes	Lipid content per oocyte	No. of oocytes	ATP content per oocyte
0	70	11	1.00 ± 0.05a	18	1.00 ± 0.03a	8	2.27 ± 0.12
1	70	11	1.17 ± 0.04b	20	0.87 ± 0.04b	11	2.64 ± 0.14

Note

Oocyte granulosa cells complexes (OGCs) were cultured in medium containing vehicle (ethanol) and 1 μmol/L DHA for 16 d. A total of 70 OGCs were used, and levels of H4K12 acetylation and amount of lipid and ATP content (pmol) per oocyte grown in vitro were examined. a,b: *P* < .05.

**FIGURE 3:**
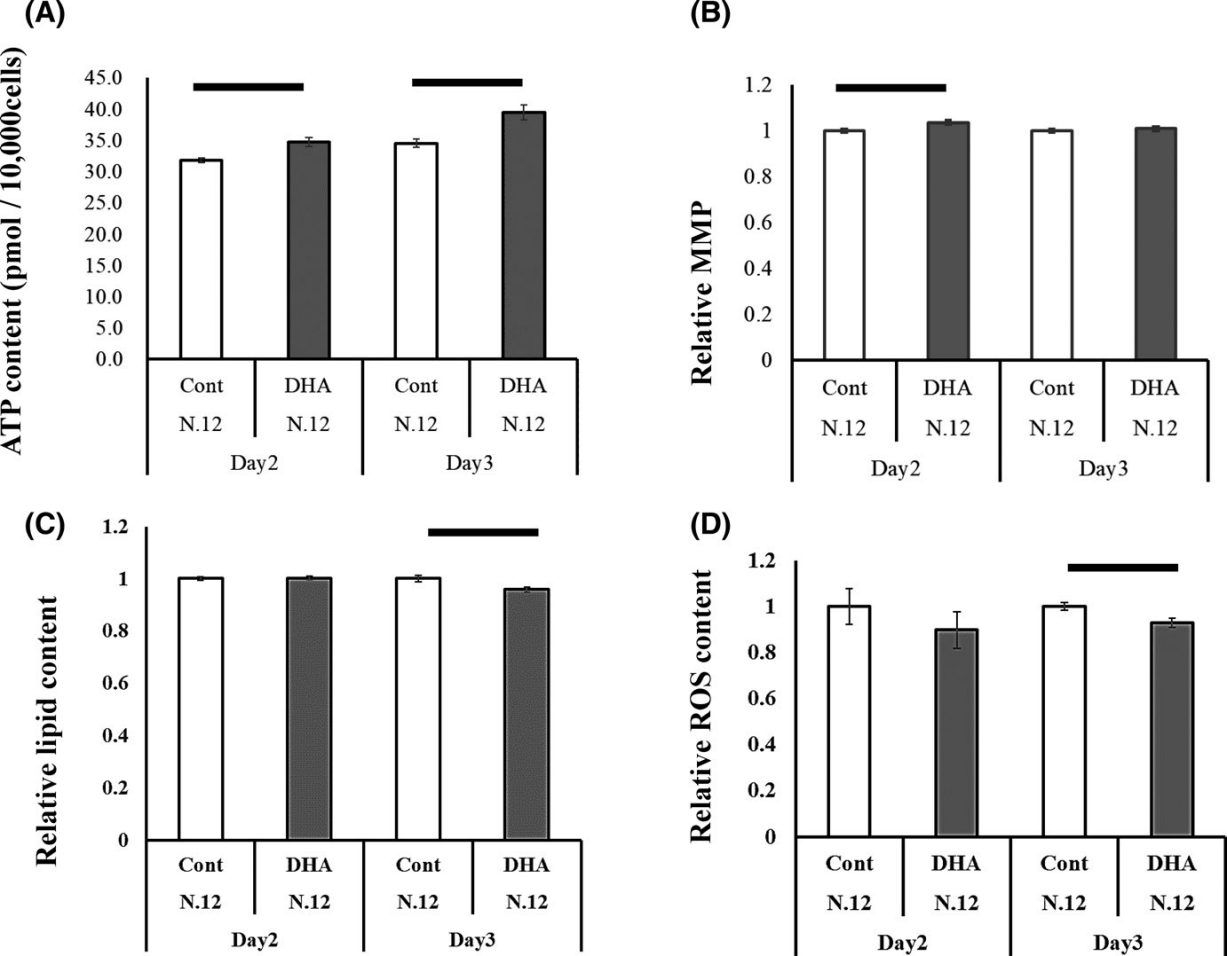
ATP, MMP, lipid content, and reactive oxygen species (ROS) content in the granulosa cells (GCs) which were cultured with or without DHA in 96‐well plate for 2 and 3 days. A, ATP content (pM) per 10,000 cells and (B) MMP of granulosa cells (GCs). Values of control GCs are defined as 1.0 for each day 2 and 3. C, Lipid content of GCs (GCs). Values of control GCs are defined as 1.0 for each day 2 and 3. D, ROS content, values of control are defined as 1.0. The experiment was repeated 12 times. Bar represents standard error mean. *P < .05

When GCs were cultured with or without DHA (1 μmol/L or vehicle) for 3 days, DHA increased the ATP content in GCs on both days 2 and 3 (Figure 3A). Furthermore, DHA increased MMP on day 2 (Figure 3B) and decreased the lipid content and ROS levels on day 3 (Figure 3C,D).

To determine the mechanism underlying the effect of DHA on GCs, we cultured GCs with or without DHA for 2 days and conducted RNA‐seq. A total of 17 457 genes were detected and 595 genes were found to be differentially expressed. The KEGG pathways enriched by the DEGs (*P* < .05) were oxidative phosphorylation, focal adhesion, and Ras signaling (Table 4). Regarding oxidative phosphorylation genes, CPT1A was significantly (*P* = .019) increased, whereas fatty acyl‐CoA synthase, CPT2, and carnitine translocase did not differ between the groups. In addition, ten of the 13 mitochondrial genes, except for COX2, ND4L, and ND5, were significantly downregulated, and of the 1139 nuclear genes encoding mitochondrial proteins, 21 genes were significantly downregulated, except for CPT1A. Genes related to mitochondrial biogenesis (PPARGC1a, NRF1, NRF2, and TFAM) did not differ between the DHA and control groups. Using the DNA extracted from the same GCs batch, we examined the mitochondrial DNA copy number in GCs but did not found a difference between the two groups (DHA, 119.6 ± 1.9 vs Vehicle, 120.2 ± 1.4, N.12).

**TABLE 4:** KEGG Pathway enriched by differential expressed genes

Kegg pathways	*P* value
Oxidative phosphorylation	1.358E‐05
Parkinson's disease	0.000144
Focal adhesion	0.002378
Ras signaling pathway	0.006352
Choline metabolism in cancer	0.006588
Alzheimer's disease	0.0182385
Glycerophospholipid metabolism	0.0213112
Platelet activation	0.0235876
Insulin resistance	0.0379412
Proteoglycans in cancer	0.0382745
mTOR signaling pathway	0.0425702
ECM‐receptor interaction	0.0450487
PI3K‐Akt signaling pathway	0.0470851

## 4 DISCUSSION

The present study demonstrated that DHA improved oocyte growth and increased their diameters and fertilization ability. Furthermore, DHA improved oocyte quality markers, including the acetylation levels of H4K12. In addition, DHA treatment reduced the lipid content in both oocytes and GCs. When GCs were cultured with DHA, lipid, and ROS levels decreased, concomitant with an increase in ATP content and MMP. RNA‐seq showed that DHA affects mitochondrial‐related genes.

The oocyte diameter increases as the follicle develops.^17^, ^18^ In cows, oocytes with a small diameter (<120 μm) have low meiotic maturation competence,^18^ whereas oocytes with a diameter greater than 120 μm had a high ability to complete nuclear maturation, fertilization, and development to term.^19^, ^20^ The quality of oocytes has been evaluated using various indexes. For example, high competent bovine oocytes have higher levels of ATP compared with those in poor quality oocytes,^21^ and competent oocytes have high acetylated histones in pigs, cows, horses, and mice.^22^, ^23^, ^24^, ^25^, ^26^ ATP and acetyl‐CoA are produced by mitochondria and are important substrates for histone acetylation.^27^ When porcine and bovine oocytes derived from EAFs were cultured in vitro, in vitro grown oocytes with high developmental competence had high levels of ATP, acetylated lysine, and acetylated H4K12.^13^, ^15^, ^28^ GCs are crucial for oocyte growth by providing energy substrates and important substrates through gap junctions. Munakata et al^14^ have reported that the number of GCs in follicles is positively related to the levels of ATP and acetylation of H4K12 in the corresponding oocytes in pigs. In addition, artificially addition of granulosa cells to bovine OGCs increased the growth of oocytes, ATP content, and the acetylation of H4K12 in oocytes grown in vitro.^14^ In the present study, we found that treatment of GCs of OGCs with DHA significantly increased the levels of ATP in the GCs. Therefore, the high ATP levels in GCs could improve the in vitro growth of oocytes by supporting high H4K12 acetylation levels.

The present study showed that DHA reduced the lipid content in both oocytes and GCs. Lipids are an important energy source for ATP production through β‐oxidation in the mitochondria during oocyte maturation.^29^ Inhibition of β‐oxidation by a carnitine palmitoyl transferase I (CPT‐1) inhibitor reduced the capacity of oocytes to develop to the blastocyst stage,^30^ whereas activation of β‐oxidation by supplementation of maturation medium with l‐carnitine improved the developmental competence of mouse oocytes.^31^ In addition, acceleration of β‐oxidation via carnitine treatment reduced ROS content and improved oocyte maturation and developmental ability.^32^ The present study showed that DHA upregulated the expression levels of CPT1A in GCs without affecting mtDNA copy number. Thus, it is suggested that DHA enhances lipid utilization in mitochondria to generate ATP, which results in a decline in lipid content.

Besides DHA‐induced high ATP and MMP and low ROS content in GCs, RNA‐seq revealed that DHA downregulated the overall expression levels of genes encoding mitochondrial proteins in both the nuclear and mitochondrial genomes. In addition, mitochondrial genome copy numbers did not differ between the two groups. Oxidative phosphorylation was the top pathway enriched by the DEGs. Supplementation of male with fish oil drastically increased DHA contents in mitochondrial membrane of skeletal muscles.^33^ DHA supplementation of the medium affects the cellular membrane ^34^ and is incorporated into the mitochondrial membrane, as determined by an increase in cardiolipin.^35^ In addition, DHA increased MMP and susceptibility of the mitochondria to oxidative stress.^36^ Therefore, it is hypothesized that DHA may change mitochondrial characteristics in GCs. The effects of DHA on mitochondria are contradictory. Although all of these studies used high concentration of DHA (50‐100 μmol/L), DHA increased mtDNA copy number and upregulated mRNA expression of genes associated with mitochondrial synthesis (PGC‐1α, NRF1, and TFAM) in mouse C2C12 myoblasts and human liver cancer cell line HepG2^37^, ^38^ and reduced ROS content and increased mitochondrial membrane potential.^38^ However, DHA decreased mitochondrial number in C2C12 myoblasts ^39^ and increased ROS content by stimulating PI3K/Akt signaling.^40^ These reports indicate that DHA has various effects on mitochondria depending on the cell type and condition. Elis et al^10^ reported that 1 μmol/L of DHA in IVM medium improved developmental ability and reduced lipid content in bovine oocytes. They addressed possible mechanism underlying the effect of DHA using microarray could not define signaling pathways.^10^ The present RNA‐seq showed enrichment in focal adhesion, Ras, mTOR, and PI3K‐Akt signaling pathways. It has been reported that treatment of colon cells with DHA reduced Ras translocalization to the plasma membrane and inhibited GTP‐bound Ras at the membrane.^35^ In addition, DHA has been shown to protect cells from palmitic acid‐induced lipotoxicity through PI3K/AKT and mTOR signaling.^41^ Furthermore, DHA induced apoptosis in cancer cells by stimulating AMPK, PI3K‐Akt, and mTOR signaling.^42^ These reports suggest that mitochondrial function, focal adhesion, Ras, PI3K/Akt, and mTOR are possible mechanisms underlying the effect of DHA on GCs. However, the evidence is not enough to determine possible mechanism of DHA on oocyte growth, and the signaling pathway needs to be elucidated in future studies.

In conclusion, consistent with previous reports on the beneficial effects of DHA on in vivo follicle development, our results indicate that DHA supports the in vitro development of oocytes derived from bovine EAFs.

## CONFLICTS OF INTEREST

The authors declare no conflicts of interest.

## AUTHOR CONTRIBUTIONS

Nagata S, Tatematsu K, Yamaguchi H, and Iwata H conducted the experiments. Inoue Yuki and Tanaka conducted gene expression analyses. Hisataka Iwata designed the study. Data analysis and drafting of the manuscript were conducted by Nagata S, Tasaki H, Koumei S, and Iwata H.

## HUMAN/ANIMAL RIGHTS

This article does not contain any studies with human. Animal study: In this study, bovine ovaries were collected from a slaughterhouse. This study was approved by the ethics committee for animal experiments of the Tokyo University of Agriculture.

## CLINICAL TRIALS REGISTRATION

This study does not include clinical trials.

## References

[1] Ruiz‐Sanz J , Pérez‐Ruiz I , Meijide S , Ferrando M , Larreategui Z , Ruiz‐Larrea M . Lower follicular n‐3 polyunsaturated fatty acid levels are associated with a better response to ovarian stimulation. J Assist Reprod Genet. 2019;36:473‐482. 30547270 10.1007/s10815-018-1384-1 PMC6439102

[2] Kermack AJ , Lowen P , Wellstead SJ , et al. Effect of a 6‐week "Mediterranean" dietary intervention on *in vitro* human embryo development: the preconception dietary supplements in assisted reproduction double‐blinded randomized controlled trial. Fertil Steril. 2020;113:260‐269. 31870562 10.1016/j.fertnstert.2019.09.041

[3] Chiu Y‐H , Karmon AE , Gaskins AJ , et al. Serum omega‐3 fatty acids and treatment outcomes among women undergoing assisted reproduction. Hum Reprod. 2018;33:156‐165. 29136189 10.1093/humrep/dex335 PMC5850735

[4] Bender K , Walsh S , Evans ACO , Fair T , Brennan L . Metabolite concentrations in follicular fluid may explain differences in fertility between heifers and lactating cows. Reproduction. 2010;139:1047‐1055. 20385782 10.1530/REP-10-0068

[5] Sinedino LDP , Honda PM , Souza LRL , et al. Effects of supplementation with docosahexaenoic acid on reproduction of dairy cows. Reproduction. 2017;153:707‐723. 28235903 10.1530/REP-16-0642

[6] Teeli AS , Sheikh PA , Patra MK , et al. Effect of dietary n‐3 polyunsaturated rich fish oil supplementation on ovarian function and interferon stimulated genes in the repeat breeding cow. Anim Reprod Sci. 2019;211:106230. 31785633 10.1016/j.anireprosci.2019.106230

[7] Elis S , Freret S , Desmarchais A , et al. Effect of a long chain n‐3 PUFA‐enriched diet on production and reproduction variables in Holstein dairy cows. Anim Reprod Sci. 2016;164:121‐132. 26651949 10.1016/j.anireprosci.2015.11.020

[8] Oseikria M , Elis S , Maillard V , Corbin E , Uzbekova S . N‐3 polyunsaturated fatty acid DHA during IVM affected oocyte developmental competence in cattle. Theriogenology. 2016;85:1625‐1634. 26898414 10.1016/j.theriogenology.2016.01.019

[9] Hoyos‐Marulanda V , Alves BS , Rosa P , et al. Effects of polyunsaturated fatty acids on the development of pig oocytes *in vitro* following parthenogenetic activation and on the lipid content of oocytes and embryos. Anim Reprod Sci. 2019;205:150‐155. 31076217 10.1016/j.anireprosci.2019.05.003

[10] Elis S , Oseikria M , Vitorino Carvalho A , et al. Docosahexaenoic acid mechanisms of action on the bovine oocyte‐cumulus complex. J Ovarian Res. 2017;10:74. 29122003 10.1186/s13048-017-0370-z PMC5679375

[11] Takahashi Y , First NL *In vitro* development of bovine one‐cell embryos: Influence of glucose, lactate, pyruvate, amino acids and vitamins. Theriogenology. 1992;37:963‐978. 16727096 10.1016/0093-691x(92)90096-a

[12] Iwata H , Hashimoto S , Ohota M , Kimura K , Shibano K , Miyake M . Effects of follicle size and electrolytes and glucose in maturation medium on nuclear maturation and developmental competence of bovine oocytes. Reproduction. 2004;127:159‐164. 15056781 10.1530/rep.1.00084

[13] Munakata Y , Kawahara‐Miki R , Shirasuna K , Kuwayama T , Iwata H . Polyacrylamide gel as a culture substrate improves *in vitro* oocyte growth from porcine early antral follicles. Mol Reprod Dev. 2017;84:44‐54. 27864905 10.1002/mrd.22758

[14] Munakata Y , Ichinose T , Ogawa K , et al. Relationship between the number of cells surrounding oocytes and energy states of oocytes. Theriogenology. 2016;86:1789‐1798. 27402087 10.1016/j.theriogenology.2016.05.036

[15] Sugiyama M , Sumiya M , Shirasuna K , Kuwayama T , Iwata H . Addition of granulosa cell mass to the culture medium of oocytes derived from early antral follicles increases oocyte growth, ATP content, and acetylation of H4K12. Zygote. 2016;24:848‐856. 27692022 10.1017/S0967199416000198

[16] Iwata H , Goto H , Tanaka H , et al. Effect of maternal age on mitochondrial DNA copy number, ATP content and IVF outcome of bovine oocytes. Reprod Fertil Dev. 2011;23:424‐432. 21426860 10.1071/RD10133

[17] Kumar A , Pandey SD . Classification of the follicle population based on oocyte diameter and number of granulosa cells in the ovary of large‐eared hedgehog, *Hemiechinus auritus* gmelin. Acta Physiol Hung. 1990;76:165‐173. 2100097

[18] Arlotto T , Schwartz JL , First NL , Leibfried‐Rutledge ML . Aspects of follicle and oocyte stage that affect *in vitro* maturation and development of bovine oocytes. Theriogenology. 1996;45:943‐956. 16727855 10.1016/0093-691x(96)00024-6

[19] Otoi T , Yamamoto K , Koyama N , Tachikawa S , Suzuki T . Bovine oocyte diameter in relation to developmental competence. Theriogenology. 1997;48:769‐774. 16728170 10.1016/s0093-691x(97)00300-2

[20] Anguita B , Jimenez‐Macedo AR , Izquierdo D , Mogas T , Paramio M . Effect of oocyte diameter on meiotic competence, embryo development, p34 (cdc2) expression and MPF activity in prepubertal goat oocytes. Theriogenology. 2007;67:526‐536. 17014901 10.1016/j.theriogenology.2006.09.003

[21] Stojkovic M , Machado SA , Stojkovic P , et al. Mitochondrial distribution and adenosine triphosphate content of bovine oocytes before and after *in vitro* maturation: correlation with morphological criteria and developmental capacity after *in vitro* fertilization and culture. Biol Reprod. 2001;64:904‐909. 11207207 10.1095/biolreprod64.3.904

[22] Endo T , Naito K , Aoki F , Kume S , Tojo H . Changes in histone modifications during *in vitro* maturation of porcine oocytes. Mol Reprod Dev. 2005;71:123‐128. 15736133 10.1002/mrd.20288

[23] Wu F‐R , Liu Y , Shang M‐B , et al. Differences in H3K4 trimethylation in *in vivo* and *in vitro* fertilization mouse preimplantation embryos. Genet Mol Res. 2012;11:1099‐1108. 22614279 10.4238/2012.April.27.9

[24] Franciosi F , Lodde V , Goudet G , et al. Changes in histone H4 acetylation during *in vivo* versus *in vitro* maturation of equine oocytes. Mol Hum Reprod. 2012;18:243‐252. 22155671 10.1093/molehr/gar077

[25] Maalouf WE , Alberio R , Campbell KHS . Differential acetylation of histone H4 lysine during development of *in vitro* fertilized, cloned and parthenogenetically activated bovine embryos. Epigenetics. 2008;3:199‐209. 18698155 10.4161/epi.3.4.6497

[26] Racedo SE , Wrenzycki C , Lepikhov K , Salamone D , Walter J , Niemann H . Epigenetic modifications and related mRNA expression during bovine oocyte *in vitro* maturation. Reprod Fertil Dev. 2009;21:738‐748. 19567217 10.1071/RD09039

[27] Morrish F , Noonan J , Perez‐Olsen C , et al. Myc‐dependent mitochondrial generation of acetyl‐CoA contributes to fatty acid biosynthesis and histone acetylation during cell cycle entry. J Biol Chem. 2010;19(285):36267‐36274. 10.1074/jbc.M110.141606 PMC2978554 20813845

[28] Shibahara H , Ishiguro A , Shirasuna K , Kuwayama T , Iwata H . Follicular factors determining the developmental competence of porcine oocyte. Reprod Med Biol. 2019;18:256‐262. 31312104 10.1002/rmb2.12269 PMC6613015

[29] Sturmey RG , Reis A , Leese HG , McEvoy TG . Role of fatty acids in energy provision during oocyte maturation and early embryo development. Reprod Domest Anim. 2009;44(Suppl 3):50‐58. 19660080 10.1111/j.1439-0531.2009.01402.x

[30] Ferguson EM , Leese HJ . A potential role for triglyceride as an energy source during bovine oocyte maturation and early embryo development. Mol Reprod Dev. 2006;73:1195‐1201. 16804881 10.1002/mrd.20494

[31] Dunning KR , Cashman K , Russell DL , Thompson JG , Norman RJ , Robker RL . Beta‐oxidation is essential for mouse oocyte developmental competence and early embryo development. Biol Reprod. 2010;83:909‐918. 20686180 10.1095/biolreprod.110.084145

[32] Somfai T , Kaneda M , Akagi S , et al. Enhancement of lipid metabolism with L‐carnitine during *in vitro* maturation improves nuclear maturation and cleavage ability of follicular porcine oocytes. Reprod Fertil Dev. 2011;23:912‐920. 21871210 10.1071/RD10339

[33] Herbst EAF , Paglialunga S , Gerling C , et al. Omega‐3 supplementation alters mitochondrial membrane composition and respiration kinetics in human skeletal muscle. J Physiol. 2014;592:1341‐1352. 24396061 10.1113/jphysiol.2013.267336 PMC3961091

[34] Nury T , Doria M , Lizard G , Vejux A . Docosahexaenoic acid attenuates mitochondrial alterations and oxidative stress leading to cell death induced by very long‐chain fatty acids in a mouse oligodendrocyte model. Int J Mol Sci. 2020;21:641. 10.3390/ijms21020641 PMC7014165 31963714

[35] Collett ED , Davidson LA , Fan YY , Lupton JR , Chapkin RS . n‐6 and n‐3 polyunsaturated fatty acids differentially modulate oncogenic Ras activation in colonocytes. Am J Physiol Cell Physiol. 2001;280:C1066‐C1075. 11287318 10.1152/ajpcell.2001.280.5.C1066

[36] Ng Y , Barhoumi R , Tjalkens RB , et al. The role of docosahexaenoic acid in mediating mitochondrial membrane lipid oxidation and apoptosis in colonocytes. Carcinogenesis. 2005;26:1914‐1921. 15975958 10.1093/carcin/bgi163 PMC4477626

[37] Lee M , Shin Y , Moon S , Kim S , Kim Y . Effects of eicosapentaenoic acid and docosahexaenoic acid on mitochondrial DNA replication and PGC‐1α gene expression in C 2 C 12 muscle cells. Prev Nutr Food Sci. 2016;21:317‐322. 28078253 10.3746/pnf.2016.21.4.317 PMC5216882

[38] Li G , Li Y , Xiao B , et al. Antioxidant activity of docosahexaenoic acid (DHA) and its regulatory roles in mitochondria. J Agric Food Chem. 2021;69:1647‐1655. 33497204 10.1021/acs.jafc.0c07751

[39] Hsueh T , Baum JI , Huang Y . Effect of eicosapentaenoic acid and docosahexaenoic acid on myogenesis and mitochondrial biosynthesis during murine skeletal muscle cell differentiation. Front Nutr. 2018;5:15. 29594127 10.3389/fnut.2018.00015 PMC5857576

[40] Tsai C‐H , Shen Y‐C , Chen H‐W , et al. Docosahexaenoic acid increases the expression of oxidative stress‐induced growth inhibitor 1 through the PI3K/Akt/Nrf2 signaling pathway in breast cancer cells. Food Chem Toxicol. 2017;108:276‐288. 28807874 10.1016/j.fct.2017.08.010

[41] Descorbeth M , Figueroa K , Serrano‐Illán M , León MD . Protective effect of docosahexaenoic acid on lipotoxicity‐mediated cell death in Schwann cells: implication of PI3K/AKT and mTORC2 pathways. Brain Behav. 2018;8:e01123. 30264903 10.1002/brb3.1123 PMC6236228

[42] Kim N , Jeong S , Jing K , et al. Docosahexaenoic acid induces cell death in human non‐small cell lung cancer cells by repressing mTOR via AMPK activation and PI3K/Akt inhibition. Biomed Res Int. 2015;2015:1‐14. 10.1155/2015/239764 PMC4538321 26339598

